# Spontaneous Resolution of an Osteochondroma

**DOI:** 10.7759/cureus.37565

**Published:** 2023-04-14

**Authors:** Joel D Badders, Kelly D Carmichael

**Affiliations:** 1 Department of Medicine, John Sealy School of Medicine, University of Texas Medical Branch, Galveston, USA; 2 Department of Orthopedic Surgery and Rehabilitation, University of Texas Medical Branch, Galveston, USA

**Keywords:** pediatrics, patellofemoral pain syndrome, pain, osteochondroma, knee, exocytosis

## Abstract

A 13-year-old girl presented with an apparent classic osteochondroma. Because she was skeletally immature, the decision was made to observe the lesion. She returned to the clinic at age 17 for unrelated reasons and was noted to no longer have the palpable mass. Magnetic resonance imaging confirmed resolution of the osteochondroma. The age range of this case fits with reported cases of childhood osteochondromas. The mechanism of resolution has been theorized to be incorporation of the lesion back into the bone during remodeling, fractures, or pseudoaneurysms. An initial period of observation is thus recommended with new patients.

## Introduction

Osteochondromas are very common skeletal lesions resulting from a herniation of part of the growth plate; they are generally not considered to be a neoplastic process. Osteochondromas grow away from the plate and have a characteristic appearance on X-ray. Solitary lesions are the norm, but cases exist of multiple osteochondromas as hereditary conditions. No standardized treatment guidelines exist, as treatment varies according to the symptoms associated with the condition. Asymptomatic cases are generally observed. Surgery is reserved for lesions that result in pain or decreased function because of their location. Thirty-one published cases of spontaneous resolution have been described [1], and the mechanism of resolution is hypothesized, but not completely known.

## Case presentation

**Figure 1:**
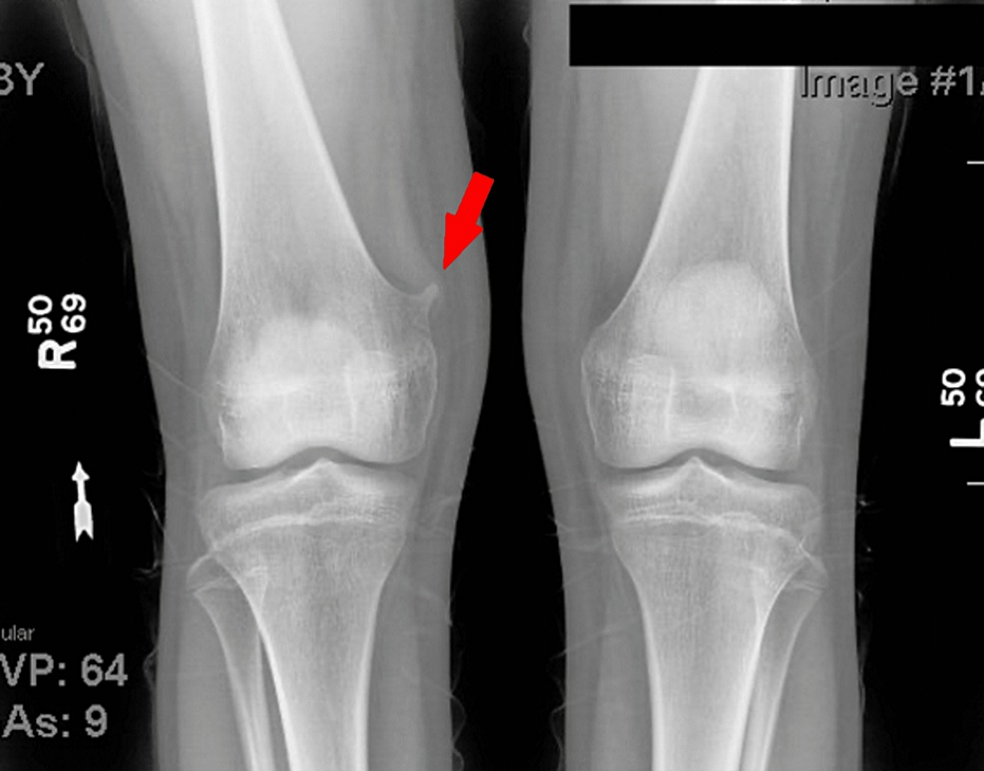
X-ray imaging revealing a bony exocytosis projecting superiorly from the right medial femoral condyle with features of an osteochondroma.

Informed consent was obtained from the patient for the publication of this case report and accompanying images. A 13-year-old girl presented to the orthopedic clinic complaining of a noticeable mass on her right knee for the past six months. She was experiencing intermittent knee pain and a “popping” feeling associated with bending the knee joint. She reported no pain on palpation. X-ray imaging revealed a bony exocytosis projecting superiorly from the right medial femoral condyle with features of an osteochondroma (Figure 1). Patellar malalignment was also noticed on radiograph, and the patient was referred to physical therapy for patellofemoral syndrome.

Seven months later, the patient returned for observation. She was tolerating activity and reported no pain. Radiographs confirmed the presence of the mass and revealed no changes in the osteochondroma. The decision was made to continue observation.

**Figure 2:**
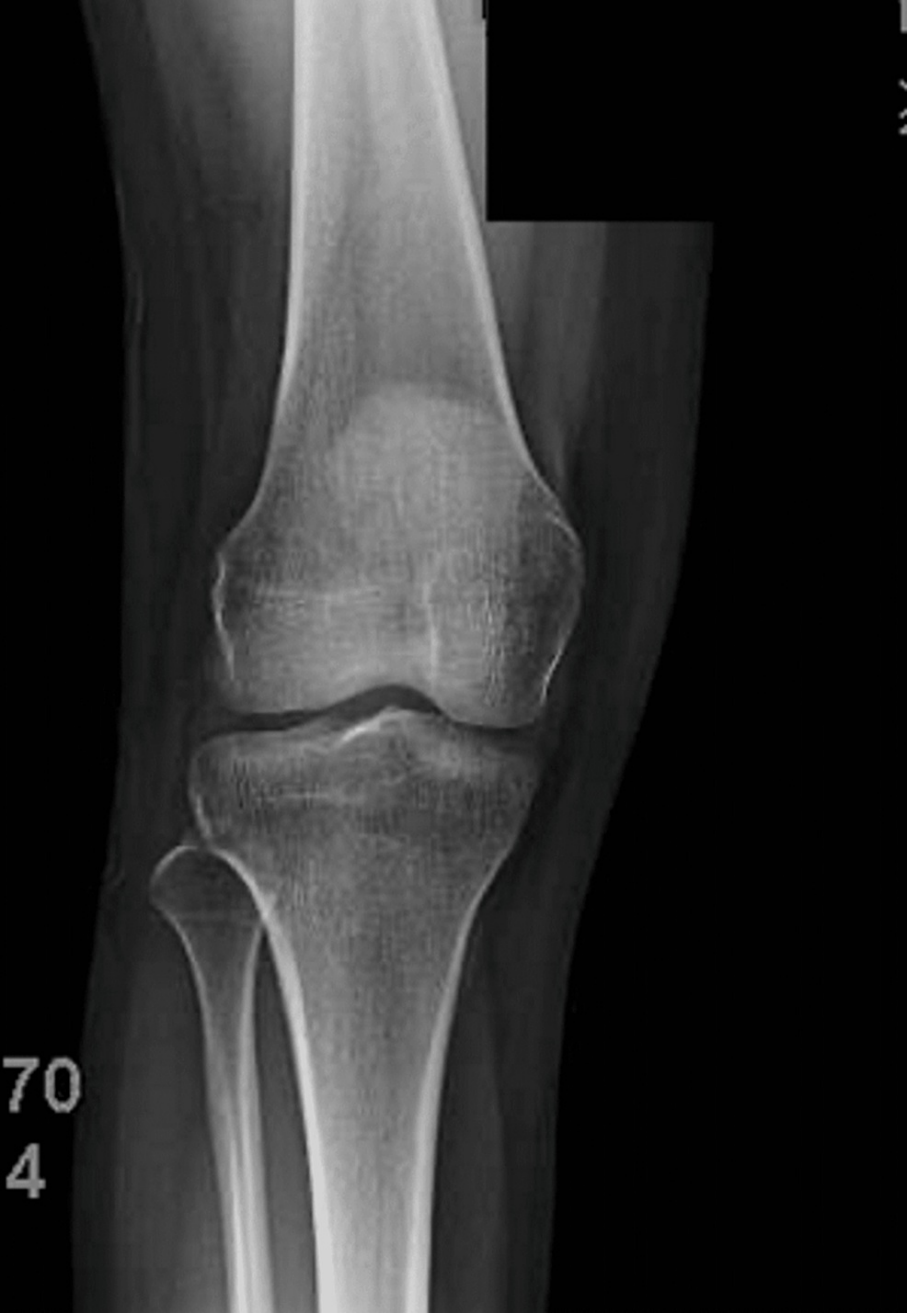
Repeat X-rays taken of the right distal femur, showing the osteochondroma to be completely resolved.

Three years later, at the age of 17, the patient returned to the clinic with right knee pain during exercise for the past two months. The pain was localized to the medial aspect of the distal femur, and she had some mechanical symptoms (i.e., popping, catching, giving out). Repeat X-rays were taken of the right distal femur (Figure 2), which showed that the osteochondroma appeared to be completely resolved.

**Figure 3:**
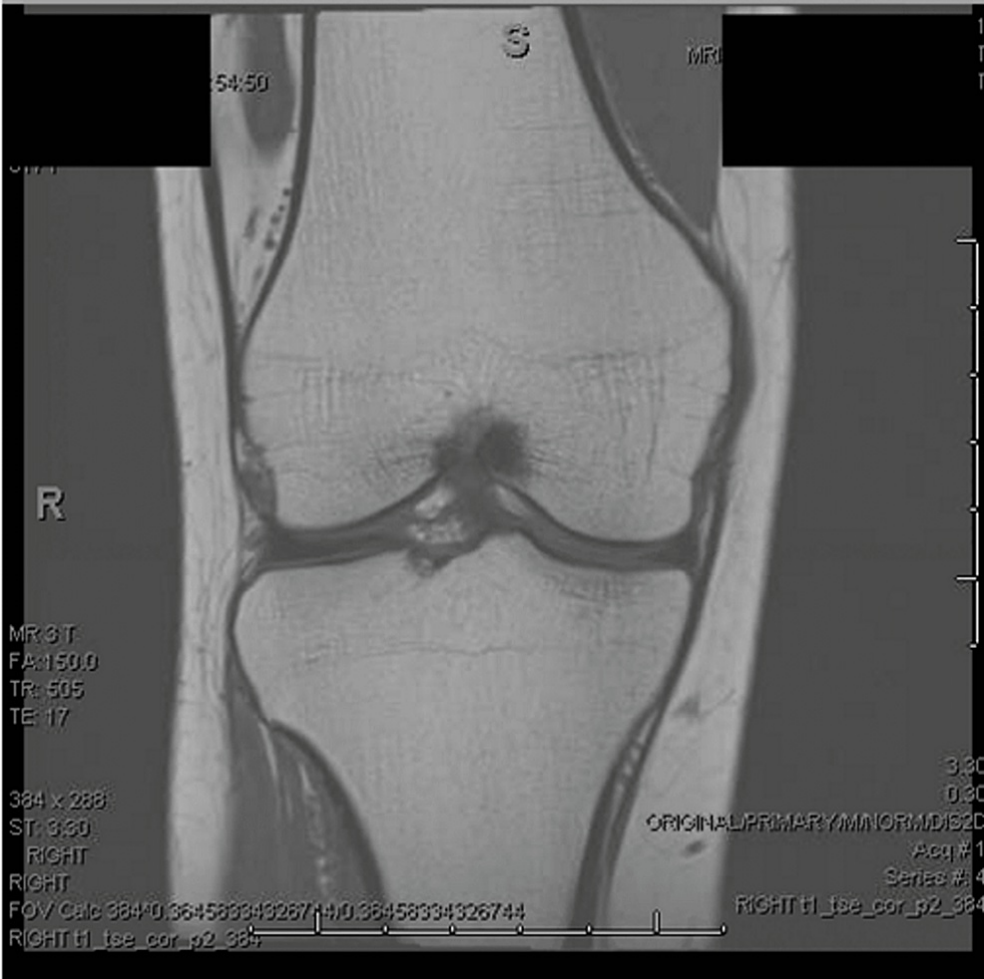
Magnetic resonance imaging performed for diagnostic purposes of the mechanical problems, confirming total resolution of the osteochondroma and no other intra-articular findings.

Magnetic resonance imaging (MRI) was performed for diagnostic purposes of the mechanical problems (Figure 3) and confirmed total resolution of the osteochondroma and no other intra-articular findings. The treatment recommended was rest, and three weeks later, the patient returned for follow-up and reported resolution of her knee pain. She returned to unrestricted activities and has not returned for additional follow-up.

## Discussion

This case demonstrated spontaneous resolution of an osteochondroma in a skeletally immature patient who presented to the clinic with mild symptoms in her right knee. An osteochondroma at the medial epicondyle of the femur was noted on X-ray. Symptoms resolved after a course of physical therapy, despite continued presence of the osteochondroma. Her last follow-up was at age 17, where the resolution was observed via X-ray and MRI. The exact mechanism for her resolution is uncertain.

Osteochondromas generally arise during childhood and account for approximately 30% of benign bone tumors. They consist of a cartilaginous cap and protrude from underlying bone, most commonly at the metaphysis [2,3]. Osteochondromas are generally treated nonoperatively. However, those associated with pain, mechanical disruption, and cosmetic disturbances may be surgically excised [4]. Osteochondromas typically increase in size during childhood. In fewer than 1% of cases, malignant transformation is noted; growth during adulthood may serve as a sign this has occurred and is considered a surgical indication [5,6]. In adulthood, patients should be counselled that tumor growth should be investigated promptly for malignancy.

Spontaneous resolution of an osteochondroma is an extraordinarily rare event, sparingly reported in the literature. Durán-Serrano et al. [1] discovered a total of 31 reported cases of resolving osteochondromas in the past 40 years. A recently published study documents how common osteochondromas may be in the general pediatric population [7]. Approximately 4.5% of the 262 asymptomatic patients (25,555 radiographs) had an osteochondroma. The subjects were followed until maturity to document normal growth and development from 1926 to 1942. None of the osteochondromas in this study resolved by the time of last radiographic follow-up.

The mechanism for resolution is under debate. Paling [3] first described a potential mechanism in 1983 by positing that growth of the osteochondroma may cease while radial growth of the bone continues. The tumor may undergo osteoclastic resorption during bone remodeling, while outward growth of the long bone subsequently incorporates the base of the tumor into its cortex [3,8]. Other described mechanisms include osteochondromas accompanied by a pressure-eroding pseudoaneurysm [8,9] and traumatic fracture of the osteochondroma, inducing bone remodeling and resorption of the lesion [1,8,10]. Paling’s theory of incorporation into the growing bone would not apply to patients who have reached skeletal maturity [3].

Paling’s theory appears to be the most applicable to our case. Our patient was 13 years old at the time of presentation, and the amount of radial growth in her bones may have been limited at the time. She reported no traumatic injuries, excluding trauma, and no pseudoaneurysm was noted at the initial or follow-up clinic visits.

Age at presentation may be the single most important factor in the potential for resolution, as the bones of younger patients will experience the most radial growth and remodeling. According to Durán-Serrano et al., 29 of the 31 reported resolutions were diagnosed before the age of 14 [1]. However, age cannot account for all resolutions; seven of the 31 cases reported documented resolution of their osteochondromas after age 17 [1,11-14], with the oldest confirmation of resolution at the age of 23 [1,14]. Of note, the age of the patients was recorded when resolution was confirmed by imaging, with resolution unnoticed and possibly occurring earlier in this group of patients.

## Conclusions

In conclusion, while spontaneous resolution of osteochondromas may be considered a rarity, the event may occur much more frequently than appreciated. Future research, increased awareness, and alertness in daily clinical practice is needed, as an unknown number of tumors may be undiagnosed and thus unreported. While not unique, this additional patient brings the published case series total to 32 for a lesion that may occur in close to 5% of children.

We recommend periodic follow-up and nonoperative management of osteochondromas in skeletally immature children who are asymptomatic or present with mild symptoms. Spontaneous resolution is a modest, yet possible, outcome in patients who have yet to reach skeletal maturity.
